# Comprehensive Echocardiographic Evaluation of Stroke in a Young Adult

**DOI:** 10.1016/j.case.2026.06.005

**Published:** 2026-08-20

**Authors:** Kevin Lipat, Harpreet Sodhi, Yekaterina Kim

**Affiliations:** a Department of Cardiology, RWJBH Newark Beth Israel Medical Center, Newark, New Jersey; b St. George's University School of Medicine, St. George's, Grenada

**Keywords:** Cardioembolic stroke, Aortic atheroma, Young adult, Papillary fibroelastoma

## Abstract

• The incidence and etiology of stroke differ across age groups.
• TEE can clarify the etiology of embolic stroke.
• Comprehensive TEE requires a systematic evaluation of cardiac and aortic structures.

## Graphical abstract

**Figure:**
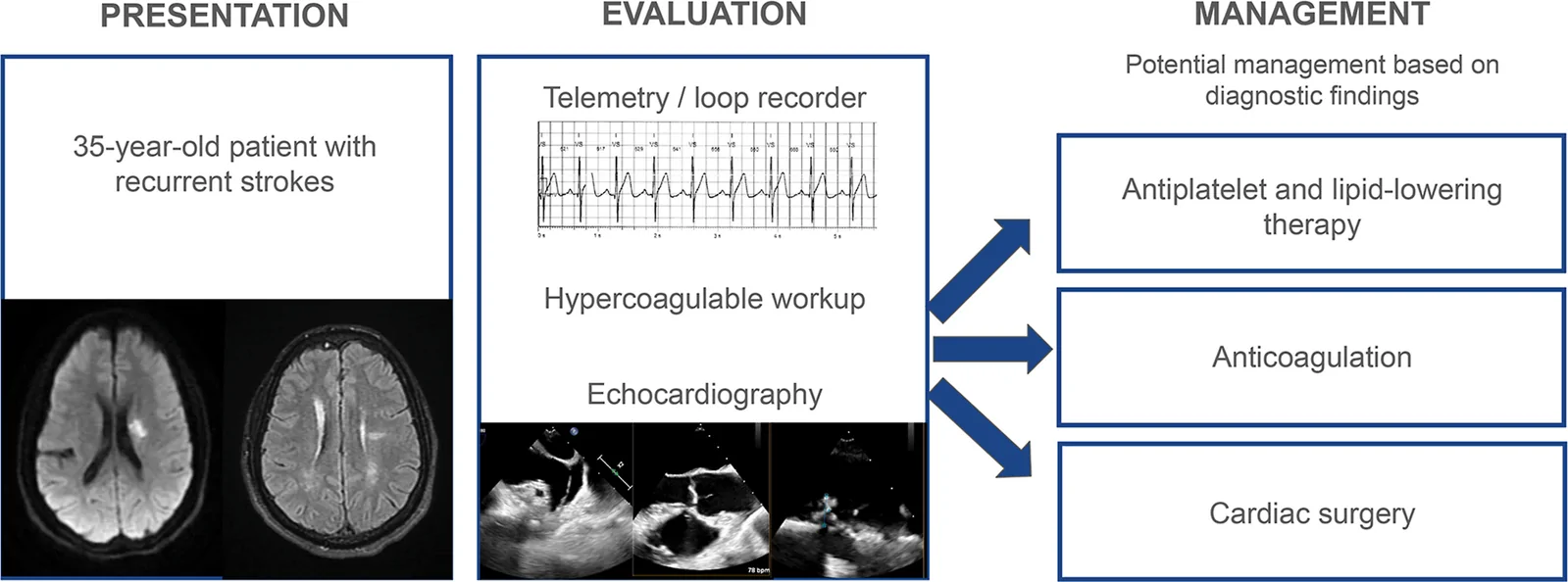

## Introduction

Stroke occurs much less commonly in young adults aged 18 to 50 years than in older populations.^1^^,^^2^ The incidence of specific etiologies of stroke also differs across age groups, with some requiring medical management such as anticoagulation and others requiring surgical solution.^1^ Echocardiographic evaluation of ischemic stroke in a young adult is often focused on identifying sources of cardioembolism such as patent foramen ovale (PFO).

We present the case of a young adult diagnosed with recurrent strokes in whom severe aortic arch atheroma was ultimately considered the most likely embolic source. We discuss the role and importance of a systematic echocardiographic evaluation for accurate diagnosis and management of ischemic stroke.

## Case Presentation

A 35-year-old patient presented to the hospital with dysarthria and right-sided facial droop, which began 18 hours before presentation and were initially transient, but returned the next day and became persistent. On arrival to the hospital, the patient’s blood pressure was 166/106 mm Hg, pulse 83 bpm, and body mass index was within the normal range (20 kg/m^2^). The patient was dysarthric with an obvious right-sided facial droop, and the remainder of the neurological examination was unremarkable.

The patient had no known past medical or surgical history and did not take any medications regularly. They reported active tobacco use, smoking 2 to 3 cigarettes daily for the past 5 years. Family history was negative for any premature coronary artery or cerebrovascular disease.

Despite the patient's young age and minimal risk factors, stroke or transient ischemic attack remained the most likely diagnoses based on the presenting symptoms and acuity of onset. Other diagnoses, such as demyelinating disease, migraine, or postictal paralysis, could be considered, but the history of present illness lacked other supporting features for these conditions.

As part of the code stroke protocol, immediate computed tomography (CT) and CT angiogram of the head and neck were performed, showing no evidence of acute infarct or intracranial hemorrhage and no significant vascular stenosis or malformation. However, there was evidence of old infarcts in both the anterior limb of the right internal capsule and the white matter of the left centrum semiovale. Subsequent magnetic resonance imaging of the brain confirmed an acute ischemic infarct in the area of the left putamen.

Routine blood investigations obtained during the hospitalization were largely unremarkable. Hemoglobin A1c was 5.8%, consistent with prediabetes. Lipid panel showed a low-density lipoprotein level of 74 mg/dL and a high-density lipoprotein level of 48 mg/dL. Activity and quantity of protein C and protein S were normal, and autoimmune workup was negative. Electrocardiogram showed normal sinus rhythm with left axis deviation, nonspecific T-wave abnormalities, and no evidence of left ventricular (LV) hypertrophy. Telemetry monitoring throughout the admission did not reveal any episodes of atrial fibrillation.

Transthoracic echocardiogram showed normal LV mass with increased relative wall thickness, consistent with concentric LV remodeling. Chamber sizes and LV ejection fraction were within normal range, and agitated saline study with Valsalva maneuver was negative for shunt. An abnormal echodensity on the aortic valve (AoV) prompted further evaluation with transesophageal echocardiogram (TEE). Transesophageal echocardiogram was performed under conscious sedation, with findings reported as possible papillary fibroelastoma (PFE) on the AoV measuring 0.3 × 0.3 cm. No large PFO was seen. An agitated saline study was performed but ultimately deemed to be nondiagnostic because of poor opacification of the right atrium, which was attributed to a low quantity of bubbles and a trajectory away from the interatrial septum (IAS).

The patient did not receive thrombolytic therapy because of delayed presentation. Treatment was guided by the neurology team and included early initiation of antiplatelet and statin therapies. Apixaban was eventually started because of suspicion for cardioembolic etiology related to the possible PFE. A loop recorder was also implanted. The patient was evaluated by the cardiothoracic surgery team before discharge, and plans were made for possible surgical intervention of the AoV after recovery from the acute stroke.

The patient was followed up with the cardiothoracic surgery team in the outpatient office. Given the recurrent strokes and finding of PFE on TEE, there was discussion about surgical resection of the PFE. On review of the TEE images, there was some uncertainty about the AoV echodensity, along with concern that the agitated saline study was nondiagnostic. Thus, a repeat TEE was requested for diagnostic clarification.

At 4 months after the index admission, repeat TEE was performed to re-evaluate the AoV. Interrogation of the AoV on 2-dimensional and 3-dimensional imaging demonstrated a small, linear echodensity arising from the AoV closure line, which appeared more consistent with Lambl's excrescences (Figure 1, Figure 2; Video 1, Video 2).

**Figure 1:**
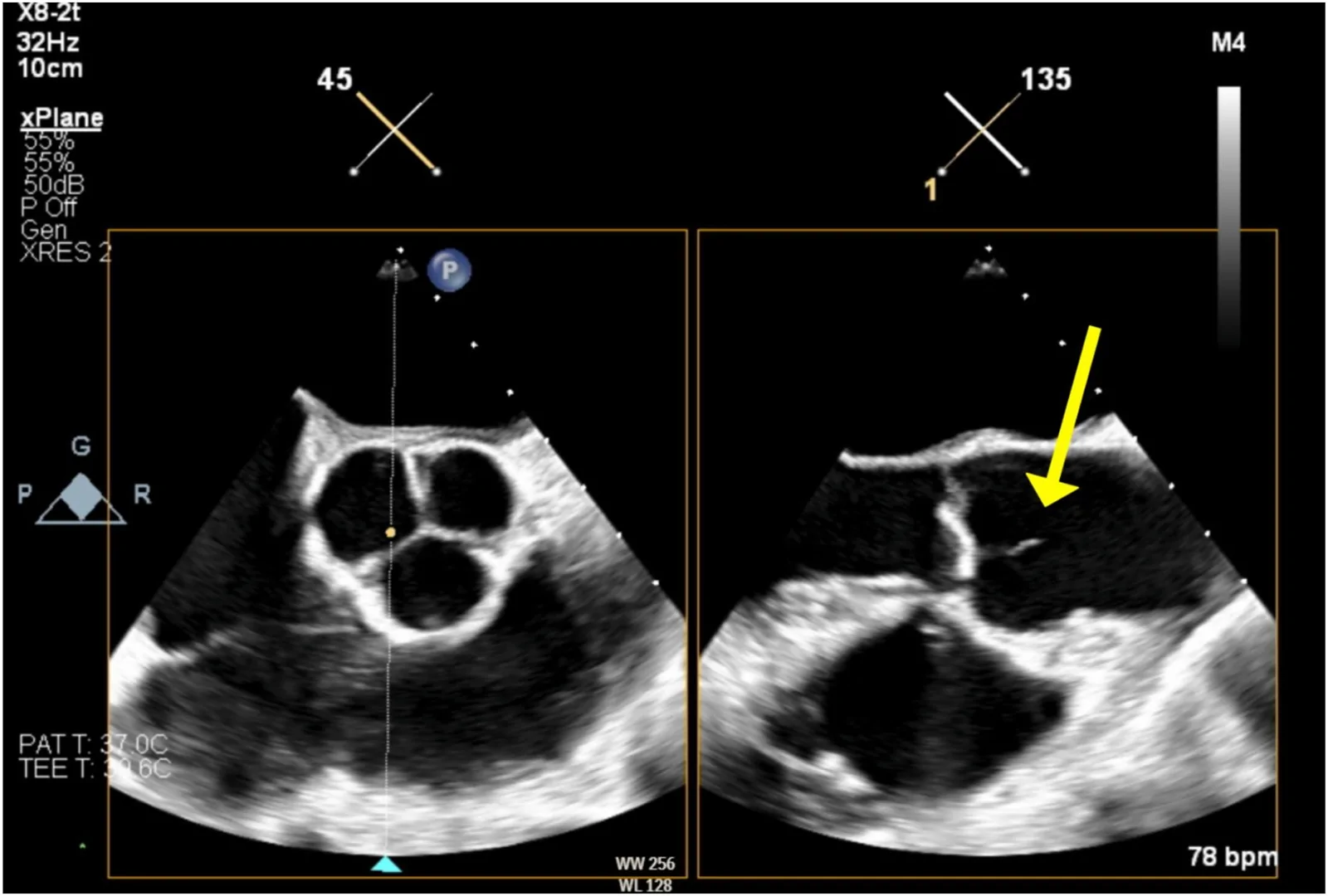
Two-dimensional transesophageal echocardiogram, midesophageal simultaneous orthogonal short-axis (left; 45°) and long-axis (right; 135°) diastolic views, demonstrates strand-like, mobile, linear filamentous masses (*arrow*) attached to the aortic valve, consistent with Lambl's excrescences.

**Figure 2:**
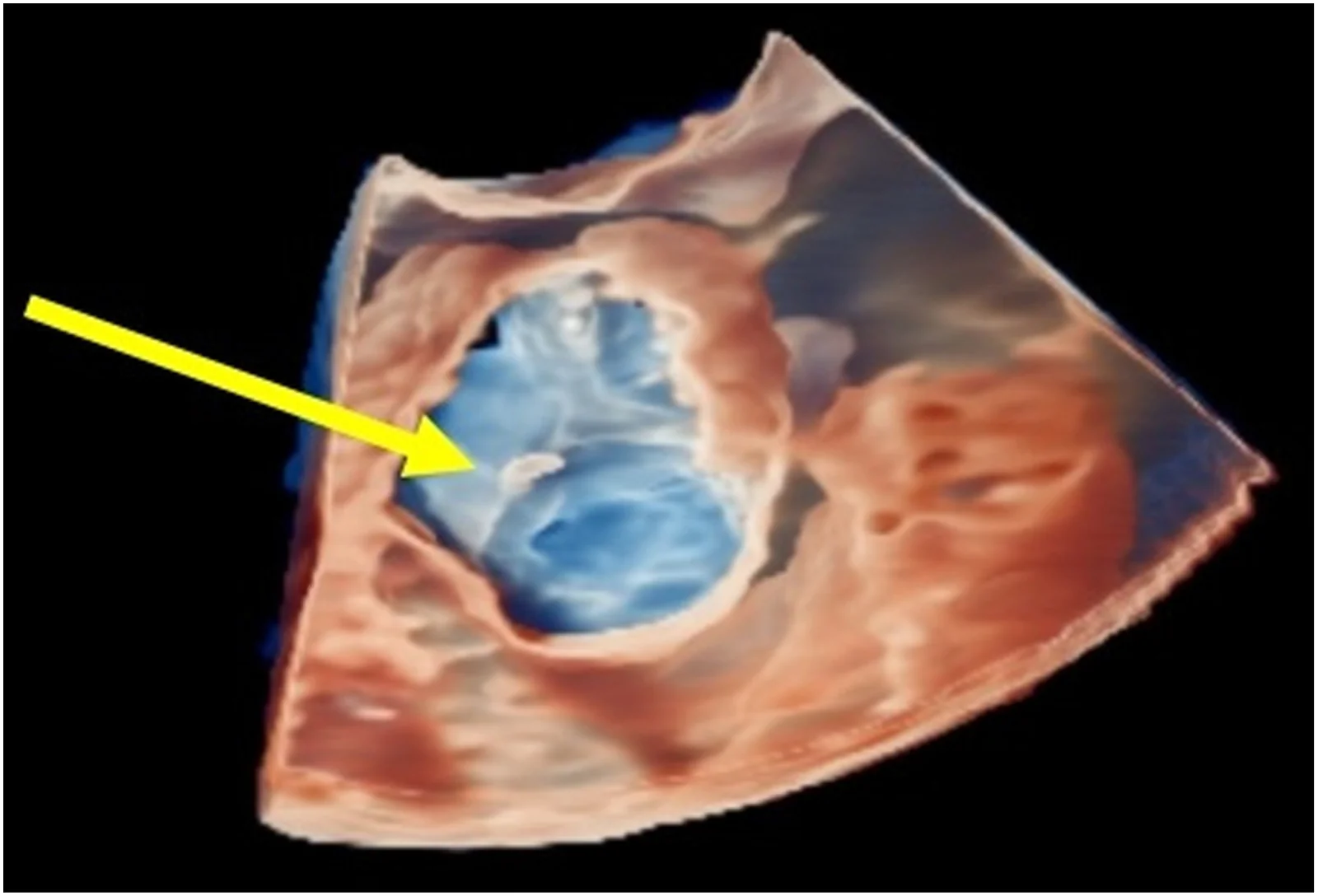
Three-dimensional transesophageal echocardiogram, volume-rendered photorealistic reconstruction of the aortic valve (AoV) in diastole, demonstrates the location of the largest Lambl's excrescences (*arrow*) arising from the AoV closure line.

Transesophageal echocardiogram was performed under conscious sedation. Agitated saline was injected via intravenous access in the lower extremity, which optimized delivery of saline bubbles from the inferior vena cava directly to the IAS (Figure 3; Video 3). The agitated saline study was negative at rest and with abdominal compression.

**Figure 3:**
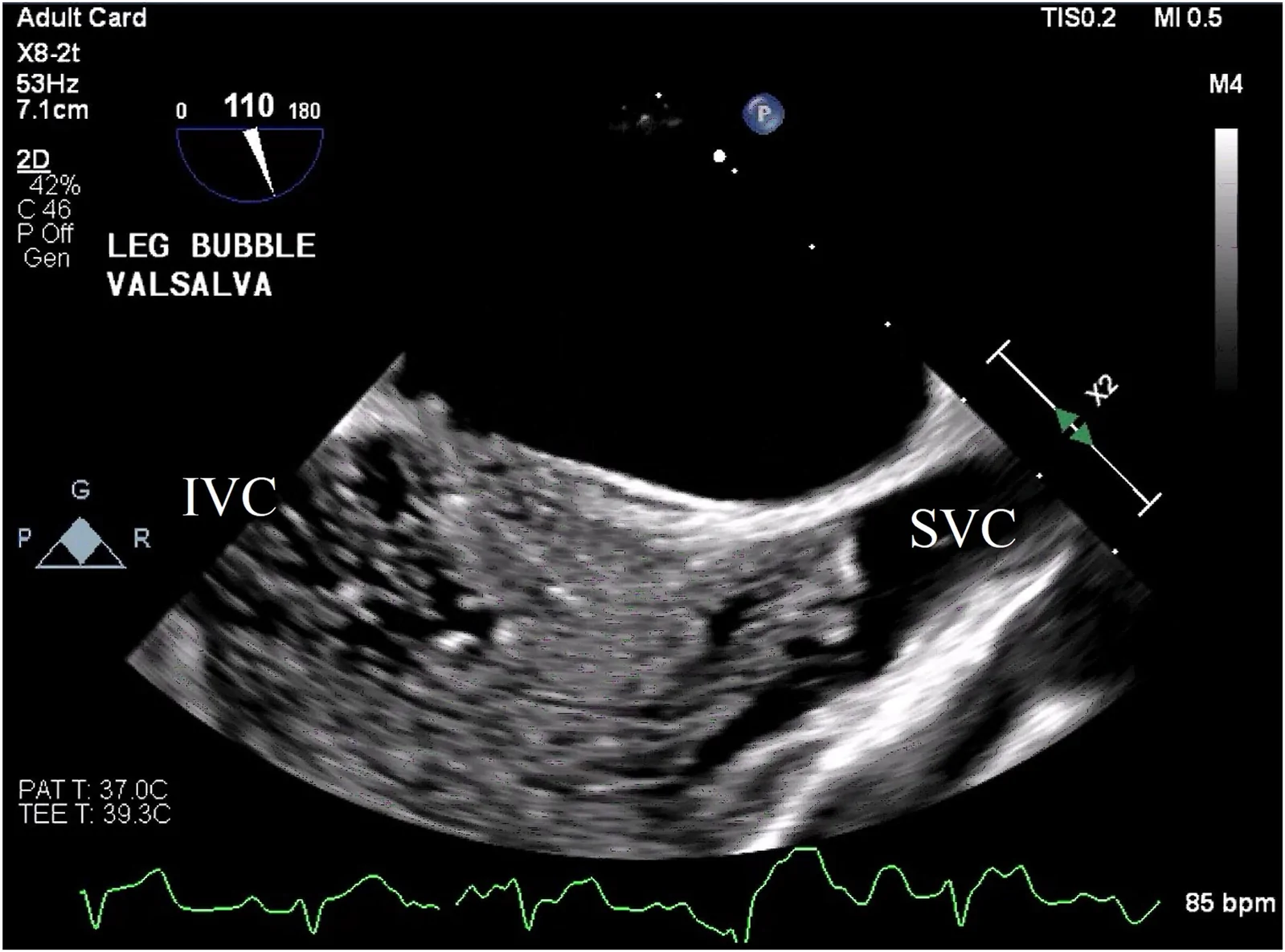
Two-dimensional transesophageal echocardiogram, midesophageal long-axis (110°) view during agitated saline study performed via lower extremity administration during Valsalva maneuver, demonstrates bubbles arriving to the right atrium from the IVC without right-to-left intracardiac shunt. *IVC,* Inferior vena cava*; SVC,* superior vena cava.

Thorough interrogation of intracardiac chambers and the left atrial appendage (LAA) did not reveal any abnormalities (Figure 4; Video 4). However, evaluation of the thoracic aorta revealed a severe, complex, grade 4 aortic atheroma along the transverse aortic arch, without clear ulceration or independently mobile thrombotic components, protruding 8 mm into the lumen (Figure 5; Video 5, Video 6).

**Figure 4:**
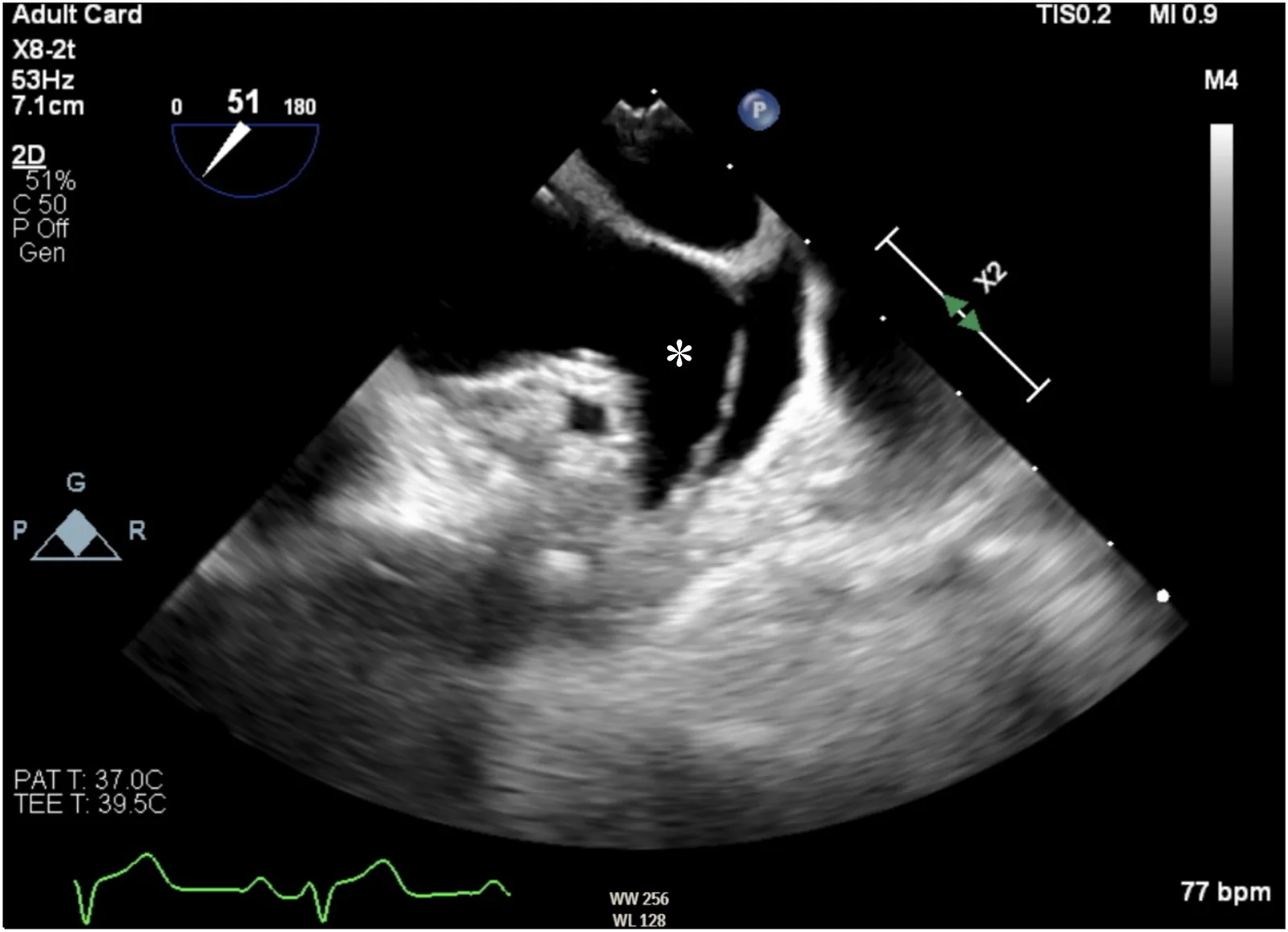
Two-dimensional transesophageal echocardiogram, midesophageal long-axis (51°) view of the left atrial appendage (*∗*), demonstrates no evidence of thrombus and a small pericardial effusion.

**Figure 5:**
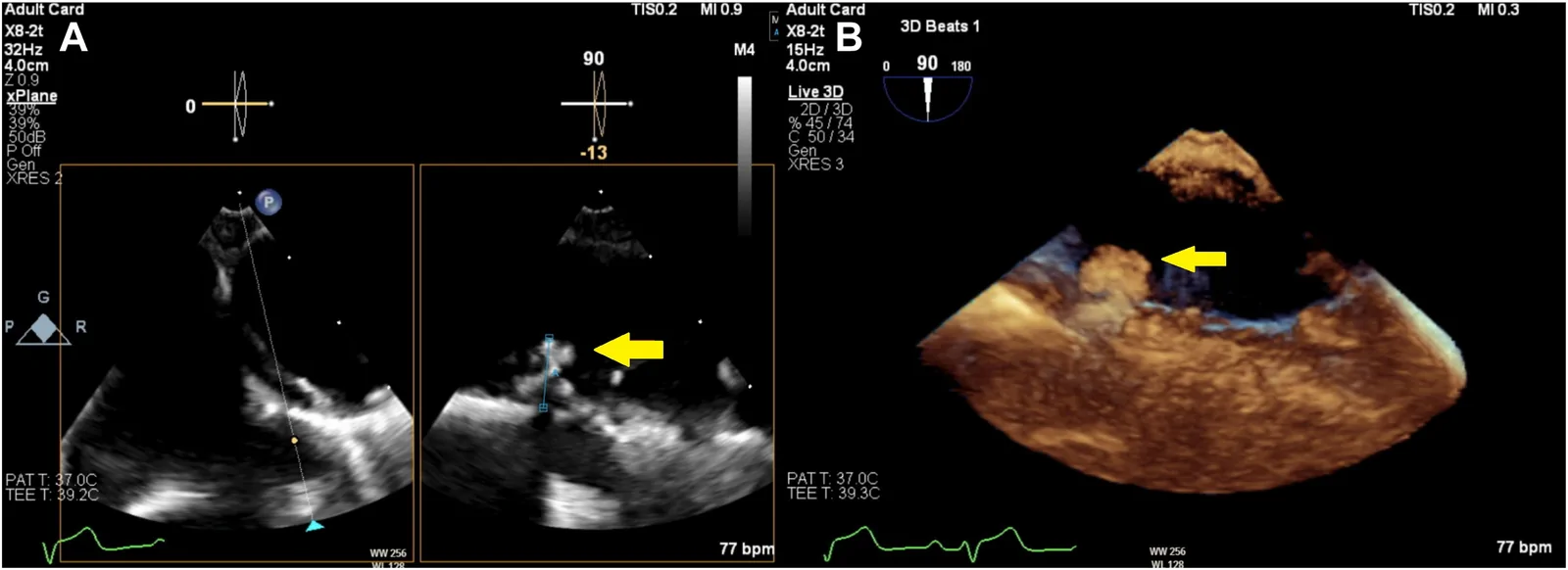
Two-dimensional transesophageal echocardiogram (TEE), upper esophageal simultaneous orthogonal short-axis (left; 0°) and long-axis (right; 90°) views of the aortic arch (**A**), aligned with the 3-dimensional TEE volume-rendered reconstruction (**B**), demonstrates a complex atheroma (*arrows*) protruding into the lumen of the aorta.

After the results of the repeat TEE, surgical intervention was deferred. At 12-month follow-up, the patient remained free of recurrent neurologic events. Serial loop recorder interrogations over 12 months did not identify atrial fibrillation. The patient continued anticoagulation and statin therapy, along with ongoing smoking cessation counseling.

## Discussion

This case illustrates the diagnostic complexity of evaluating ischemic stroke in a young adult when serial imaging identifies more than one potential embolic source. Initial TEE raised concern for PFE, which prompted consideration of surgical intervention. However, repeat TEE with systematic reassessment of the AoV, IAS, LAA, and thoracic aorta demonstrated findings more consistent with Lambl's excrescences and identified a severe protruding aortic arch atheroma. The aortic lesion was, therefore, considered the most likely embolic source.

The initial diagnostic concern centered on the AoV echodensity. A PFE is an important consideration in patients with embolic stroke because of its association with systemic embolization and its potential surgical management.^1^^,^^3^^,^^4^ In this patient, however, repeat 2-dimensional and 3-dimensional TEE demonstrated small, linear, mobile echodensity arising from the valve closure line, favoring Lambl's excrescences rather than a discrete pedunculated valvular tumor.^1^^,^^5^ This distinction was clinically important because the initial interpretation had led to discussion of AoV surgery, whereas the repeat study shifted management toward medical therapy. The repeat examination also benefited from dedicated reassessment of valve morphology and a more comprehensive interrogation of the thoracic aorta.

Assessment for paradoxical embolism is also essential in a young patient with stroke. The initial TEE agitated saline study was limited by poor right atrial opacification and, therefore, was not considered diagnostic. In addition, TEE performed under conscious sedation may reduce the effectiveness of Valsalva maneuver and lower sensitivity for PFO detection.^6^ For this reason, the repeat TEE included agitated saline injection through lower extremity venous access, allowing bubbles to enter the right atrium from the inferior vena cava and better direct contrast toward the IAS.^7^^,^^8^ No right-to-left shunt was observed at rest or with abdominal compression. Nevertheless, a very small PFO cannot be excluded with absolute certainty, and paradoxical embolism remains a less likely but possible alternative mechanism.

The most clinically significant finding on repeat TEE was the severe protruding aortic arch atheroma measuring 8 mm. Aortic arch atheroma is a recognized source of cerebral embolism, particularly when plaque thickness exceeds 4 mm or when mobile, ulcerated, or thrombotic components are present.^1^^,^^9^ In this patient, the size and protruding morphology of the plaque made it a plausible embolic source, despite the patient's young age and limited traditional risk factors. Its identification also highlights a potential limitation of the initial stroke evaluation: depending on the CT angiography protocol, the aortic arch may not be fully visualized or carefully assessed. Dedicated echocardiographic interrogation of the thoracic aorta was, therefore, essential in this case.

Retrospective review of the initial CT angiography demonstrated a corresponding aortic arch calcified atherosclerotic plaque, suggesting that the lesion was present at the time of index presentation but was not initially recognized as a potential embolic source (Figure 6).

**Figure 6:**
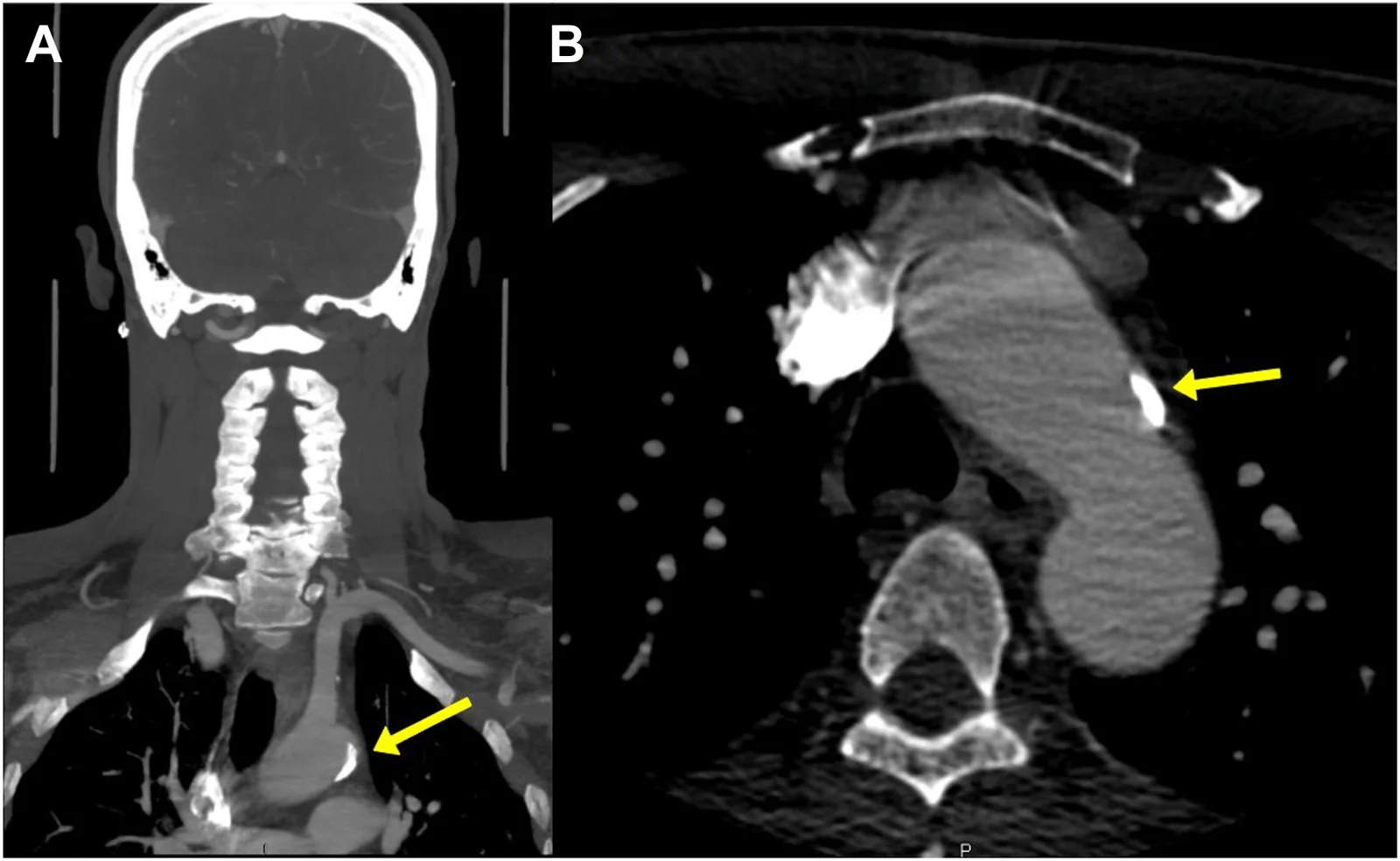
Computed tomography angiography of the head and neck with (**A**) coronal and (**B**) axial views of the aortic arch atheroma (*arrows*).

The presence of severe aortic atheroma in a 35-year-old patient is unusual. Active tobacco use and prediabetes may have contributed, although the degree of plaque burden remained disproportionate to the apparent risk profile. The differential diagnosis for an aortic mass in a young patient also includes thrombus, complex atheroma with superimposed thrombotic material, vegetation, tumor, and, less commonly, inflammatory or vasculitic disease.^10^^,^^11^ In this case, the echocardiographic appearance and location favored complex atheroma, and autoimmune and hypercoagulability testing did not reveal an alternative systemic explanation.

Management was guided by the revised interpretation of the repeat TEE. Because the valvular finding was favored to represent Lambl's excrescences rather than PFE, surgical intervention was deferred. The patient was managed medically with anticoagulation, statin therapy, and lifestyle modification, including smoking cessation counseling. Although the optimal antithrombotic strategy for complex aortic arch atheroma remains uncertain, medical therapy was favored given the absence of a definite surgical target and the presence of a high-risk aortic plaque.^10^ Implantable loop recorder monitoring did not reveal atrial fibrillation during follow-up, making occult atrial arrhythmia a less likely competing mechanism.

This case emphasizes the risk of premature diagnostic closure during stroke evaluation in young adults. Although evaluation for PFO is often prioritized in this population, echocardiographic evaluation should not stop after IAS assessment. A systematic TEE approach should include careful evaluation of the valves, left atrium and LAA, ventricular chambers, IAS, and thoracic aorta.^1^ In this patient, repeat comprehensive imaging changed the presumed diagnosis, avoided potential valve surgery, and redirected management toward treatment of a severe aortic arch atheroma.

## Conclusion

This case highlights the importance of systematic echocardiographic evaluation in young patients with ischemic stroke, particularly when multiple potential embolic sources are identified. Comprehensive reassessment with repeat TEE altered the presumed diagnosis, avoided unnecessary surgical intervention, and identified severe aortic arch atheroma as the most likely embolic source.

## Ethics Statement

The authors declare that the work described has been carried out in accordance with The Code of Ethics of the World Medical Association (Declaration of Helsinki) for experiments involving humans.

## Consent Statement

Complete written informed consent was obtained from the patient (or appropriate parent, guardian, or power of attorney) for the publication of this study and accompanying images.

## Funding Statement

The authors declare that this report did not receive any specific grant from funding agencies in the public, commercial, or not-for-profit sectors.

## Disclosure Statement

The authors reported no actual or potential conflicts of interest relative to this document.

## Supplementary Data

Video 1 Two-dimensional transesophageal echocardiogram, midesophageal simultaneous orthogonal short-axis (left; 45°) and long-axis (right; 135°) views, demonstrates strand-like, mobile, linear filamentous masses attached to the aortic valve, consistent with Lambl's excrescences.

Video 2 Three-dimensional transesophageal echocardiogram, volume-rendered photorealistic reconstruction of the AoV, demonstrates a normal, trileaflet aortic valve (AoV) with the Lambl's excrescences arising from the AoV closure line.

Video 3 Two-dimensional transesophageal echocardiogram, midesophageal long-axis (110°) view during agitated saline contrast study performed via lower extremity administration during Valsalva maneuver, demonstrates bubbles arriving in the right atrium from the inferior vena cava without right-to-left intracardiac shunt.

Video 4 Two-dimensional transesophageal echocardiogram, midesophageal long-axis (51°) view of the left atrial appendage, demonstrates no evidence of thrombus and a small pericardial effusion.

Video 5 Two-dimensional transesophageal echocardiogram, upper esophageal simultaneous orthogonal short-axis (left; 0°) and long-axis (right; 90°) views of the aortic arch, demonstrates the complex atheroma protruding into the lumen of the aorta.

Video 6 Three-dimensional transesophageal echocardiogram, upper esophageal volume-rendered long-axis (A) and en face (B) reconstruction views of the aortic arch, demonstrates the complex atheroma protruding into the lumen of the aorta.
